# The Association Between Inflammatory Bowel Disease and Exposure to Tobacco Smoking: A Case-Control Study in Qatar

**DOI:** 10.2147/IJGM.S393284

**Published:** 2023-01-21

**Authors:** Bushra Abdallah, Mariah Arif, Maryam Al-Malki, Razan Hourani, Tamader Al-Maadeed, Nidal Khodr, Ghaith Al-Kuwari, Mashael Al-Siddiqi, Tanya Kane, Tawanda Chivese

**Affiliations:** 1 Department of Population Medicine, College of Medicine, QU Health, Qatar University, Doha, Qatar

**Keywords:** Crohn’s disease, ulcerative colitis, inflammatory bowel disease, smoking, childhood passive smoking, Middle East and North Africa

## Abstract

**Objective:**

This research investigated the association between childhood and adulthood tobacco smoking exposure with ulcerative colitis (UC) and Crohn’s disease (CD) in Qatar.

**Study Design and Setting:**

In this case-control study, CD and UC cases were matched to controls of the same age and sex. The associations between UC and CD and childhood passive smoking and adulthood active smoking were assessed using conditional multivariable logistic regression.

**Results:**

A total of 89 CD cases, median age of 37 years (IQR 29–47), and 362 UC cases, median age of 35 years (IQR 28–44), and equal numbers of controls were included. After multivariable logistic regression, CD was associated with higher odds of being a current smoker (OR 2.51, 95% CI 0.85–7.37, p=0.095), with weak evidence against the null hypothesis. This association was more pronounced in women, where CD was associated with both adulthood current smoking (OR 42.71, 95% CI, 1.17–1559.57, p=0.041), and childhood smoking exposure (OR 8.23, 95% CI, 1.36–49.66, p=0.021). In males, no associations were observed between CD and the smoking exposures. No associations were observed between UC and both smoking variables.

**Conclusion:**

In Qatar, adulthood tobacco smoking appears to increase the odds of CD. Further, our findings suggest that both childhood and adulthood cigarette smoke exposure may possibly have a detrimental effect on the odds of CD in females but not in males, although further research is needed.

## Introduction

Inflammatory bowel disease (IBD) encompasses many chronic gastrointestinal diseases, including Ulcerative Colitis (UC) and Crohn’s Disease (CD).^1^ Although the prevalence of IBD appears to be flattening in Western Europe and North America, at around 0.3%, limited data indicates that IBD is increasing in low and middle-income countries.^2^ In the Middle East and North Africa (MENA), the prevalence of IBD is largely unknown and data about possible risk factors of IBD are also scarce. However, findings, mostly pertaining to UC, from a few countries suggest a rising incidence of IBD in the region.^3–5^ Data related to CD are also scarce. There appears to be no published data on either UC or CD from Qatar.

CD and UC are multifactorial diseases whose causes are not completely understood. Research suggests that these diseases may result from the interplay between genetic susceptibility, innate and adaptive immunity, dysbiosis, and environmental factors.^1^ However, the contribution of each of these factors is still unknown. Some of the environmental factors that have been identified as risk factors for UC and CD include diet, UV exposure, stress, and smoking, although their contribution to disease risk differs between CD and UC.^1^ Recent research also suggests that the effects of some of these risk factors on the risk of UC and CD, especially smoking, may differ between populations^6^ and between genders.^7^

Tobacco smoking is one of the most researched risk factors for IBD development. Substantial research has shown that adulthood smoking is associated with a higher risk of CD.^8–10^ However, this association remains debatable as other studies have not found smoking to be a risk factor for CD development.^11^,^12^ For example, in a study of Ashkenazi Jews, a population with one of the highest prevalence rates of CD, smoking has not been shown to be a risk factor.^12^ Similarly, in Spain, no differences were observed in smoking exposure between CD cases and controls.^11^ These findings suggest that the effect of smoking on CD development may differ across populations. On the other hand, while only a few studies have been conducted, most findings point towards weak or no associations between childhood passive smoking and CD.^8^,^13^ To the best of our knowledge, research on the association between cigarette smoking and CD in Arab populations in the Middle East and North Africa are scarce.

A paradoxical association has been demonstrated between tobacco smoking and UC.^14^ A meta-analysis established that, in comparison to former or non-smokers, UC was less likely to develop in active smokers.^8^ It is still unclear why tobacco smoking may have a protective effect on UC, although some research suggests that the nicotine in tobacco may have an anti-inflammatory effect by increasing mucin production and decreasing proinflammatory cytokine expression.^14^ However, although most of the findings have pointed towards a protective effect of smoking on UC risk, some studies have not found smoking to be protective against the risk of UC.^15^,^16^ This may be due to inter-study differences in sampling, design and methods, and perhaps may also suggest a possible difference in the effect of smoking on UC in different populations. Similar to CD, research on the association between childhood passive smoking and the risk of UC is also scarce in Arabic populations.

This study aimed to contribute to the under-researched area of the etiology of IBD in the Middle East and North Africa region, by studying the association between UC and CD with exposure to tobacco smoking in both childhood and adulthood. Specifically, we investigated whether either UC or CD were associated with exposure to childhood passive smoking and adulthood active tobacco smoking in Qatar. We also investigated these associations stratified by gender.

## Materials and Methods

### Design and Setting

This was a case-control study, conducted between September 2021 and September 2022, in which participant data were obtained from the Qatar Biobank (QBB), a repository of biological samples from the population of Qatar. The QBB is an agnostic-hypothesis based prospective longitudinal cohort, that recruits long term residents and citizens of Qatar and collects data from biological samples, questionnaires, omics and imaging data.^17^ Thus, individuals with and without any exposures and any diseases are eligible to participate in the QBB cohort as long as they satisfy the long-term residency and citizenry status for follow-up purposes. For this sub-study, individuals were included if they were aged 18 years or older, had complete data on IBD disease status, complete data on childhood and adult tobacco smoking exposure, and confounders of interest.

Cases were individuals who reported a pre-existing diagnosis of either UC or CD. All the cases with either CD or UC were identified from the QBB and included in this study. In clinical practice, UC and CD are diagnosed using the guidelines set out by the European Crohn’s and Colitis Organization (ECCO).^18^ The ECCO guidelines require biochemistry, imaging studies, endoscopy, biopsies, blood tests, and stool examinations in an individual with a compatible clinical presentation. A diagnosis of UC requires at least two biopsies of the inflamed region and possibly more biopsies from uninflamed regions of the colon. For CD, a diagnosis requires a minimum of three histological features, which may include, crypt abnormalities with mucin depletion, granulomas, and mucin preservation in the inflamed sites.^18^ Based on the eligibility criteria, 362 UC cases and 89 CD cases were included in the study.

Controls were individuals without IBD, specifically UC, CD, irritable bowel syndrome or indeterminate colitis, and without any known diseases of the immune system such as myasthenia gravis, systemic sclerosis, rheumatoid arthritis, lupus, multiple sclerosis, type 1 diabetes, Graves’ Disease and psoriasis. Controls were matched to cases for age, using a 5-year window and sex on a one-to-one basis, bringing the total number of study participants to 902. As this was an unfunded study, the maximum participants that the QBB avails is 1000, and therefore no attempt was made to increase the case: control matching ratio.

### Data Collection and Variables

This study uses data that were collected by the QBB, and therefore this section describes the data collection procedures that were used by the QBB. A self-administered questionnaire was utilized at the QBB to obtain data on; 1) participant demographics; 2) second-hand passive smoke exposure during childhood, and 3) smoking status during adulthood. Childhood exposure to passive smoking was defined as the presence of at least one parent smoking in the household during the participant’s early childhood. Active smoking was defined as the consumption of cigarette smoke during adulthood. Participants were stratified according to their reported smoking status into four groups; 1) never smoked; 2) former smoker; 3) current smoker; and 4) ever smoker. A current smoker was defined as a person who smokes daily or occasionally during the week. An ever-smoker was defined as either a current or former smoker.

### Statistical Analysis

Numerical data were assessed for normality using histograms and the D’Agostino-Pearson Test. The numerical data were summarised as mean and standard deviation if normally distributed, or median and interquartile range if not normally distributed. Student’s independent groups t-tests were used to compare groups for normally distributed numerical data, and the Wilcoxon Rank Sum test was used for skewed data. Categorical data were presented as frequencies and percentages, and the Chi-square test used to test hypotheses and compare categorical data between cases and controls.

The association between IBD and exposure to childhood and adulthood factors was evaluated using conditional multivariable logistic regression analysis while adjusting for potential confounders using two models. The first model assessed the association between UC and exposure to childhood passive smoking, adulthood smoking status, and ever-smoking status. The confounders adjusted for were nationality, education and income. In addition, childhood passive smoking and adulthood smoking status were included in the same model. The second model assessed the association between CD and the above-mentioned exposures. In this model, both childhood passive smoking and adulthood smoking status exposures were included together, and adjusted for nationality, income, and education. Odds ratios (OR) were reported for the conditional multivariable logistic regression models. Conditional logistic regression was used as the cases and controls were matched for age and sex. The analysis was repeated, stratified by gender. We reported 95% confidence intervals (95% CIs) of the ORs, and the exact p values were reported and interpreted as evidence against the null hypothesis. All conditional logistic regression models were tested for link specification using the *linktest* and model fit using McFadden’s test. All the statistical analyses were carried out using STATA 17.0.

### Ethical Considerations

Written, informed consent was obtained by QBB from all participants before data collection. This study received IRB approval from the QBB (Ref - Ex-2022-QF-QBB-RES- ACC-00071-0194). This study also received a waiver of ethics review from the Qatar University Institutional Review Board (Ref – QU-IRB 1755-E/22).

## Results

At the time of this study, there were a total of 89 cases of CD and 362 cases of UC in the QBB. These cases were then matched to an equal number of controls resulting in a total of 902 participants who were included in this study.

The demographic characteristics of the participants are shown in Tables 1 and 2. The median age was 35 years (IQR 28–44) and 37 years (IQR 29–47) among UC and CD, respectively. Among all the cases and controls, most of the participants were of Qatari nationality (> 80%). Of the cases, about two-thirds of those with UC were female (63%), while just over half of those with CD were male (55%).

**Table 1:** Comparison of Demographic Characteristics Between Ulcerative Colitis Cases and Controls

Factor	Level	UC Control	UC Cases	*p*-value
N		362	362	
Age, median (IQR)		35.0 (28.0, 44.0)	35.0 (28.0, 44.0)	0.98
Gender	Male	134 (37.0%)	134 (37.0%)	1.00
	Female	228 (63.0%)	228 (63.0%)	
Nationality	Qatari	292 (80.7%)	298 (82.8%)	0.46
	Non-Qatari	70 (19.3%)	62 (17.2%)	
BMI, mean (SD)		30.1 (6.9)	28.9 (5.9)	0.009
Education	Primary & secondary	171 (47.2%)	207 (57.2%)	0.016
	Undergraduate	170 (47.0%)	143 (39.5%)	
	Postgraduate	21 (5.8%)	12 (3.3%)	
Employment	Employed	240 (66.3%)	247 (68.2%)	0.58
	Unemployed	122 (33.7%)	115 (31.8%)	
Income	<10,000	84 (23.3%)	74 (20.4%)	0.48
	10,000–80,000	235 (65.1%)	246 (68.0%)	
	>80,000	8 (2.2%)	13 (3.6%)	
	Do not know	34 (9.4%)	29 (8.0%)	
Hypertension	No	310 (86.1%)	328 (90.6%)	0.060
	Yes	50 (13.9%)	34 (9.4%)	
Diabetes Mellitus	No	301 (83.6%)	322 (89.0%)	0.037
	Yes	59 (16.4%)	40 (11.0%)	
Hypercholesterolemia	No	278 (77.2%)	291 (80.4%)	0.30
	Yes	82 (22.8%)	71 (19.6%)

**Table 2:** Comparison of Demographic Characteristics Between Crohn’s Disease Cases and Controls

Factor	Level	CD Control	CD Cases	*p*-value
N		89	89	
Age, median (IQR)		37.0 (29.0, 47.0)	37.0 (29.0, 47.0)	1.00
Gender	Male	49 (55%)	49 (55%)	1.00
	Female	40 (45%)	40 (45%)	
Nationality	Qatari	71 (80%)	74 (83%)	0.56
	Non-Qatari	18 (20%)	15 (17%)	
BMI, mean (SD)		32.0 (6.9)	28.5 (5.3)	<0.001
Education	Primary & secondary	40 (45%)	41 (47%)	0.70
	Undergraduate	41 (46%)	42 (48%)	
	Postgraduate	8 (9%)	5 (6%)	
Employment	Employed	63 (71%)	64 (73%)	0.77
	Unemployed	26 (29%)	24 (27%)	
Income	<10,000	26 (29%)	16 (18%)	0.18
	10,000–80,000	56 (63%)	59 (66%)	
	>80,000	2 (2%)	3 (3%)	
	Do not know	5 (6%)	11 (12%)	
Hypertension	No	74 (83%)	80 (90%)	0.19
	Yes	15 (17%)	9 (10%)	
Diabetes Mellitus	No	75 (84%)	80 (90%)	0.26
	Yes	14 (16%)	9 (10%)	
Hypercholesterolemia	No	62 (70%)	70 (79%)	0.17
	Yes	27 (30%)	19 (21%)	

### The Association Between Smoking Exposure and Ulcerative Colitis

**Figure 1:**
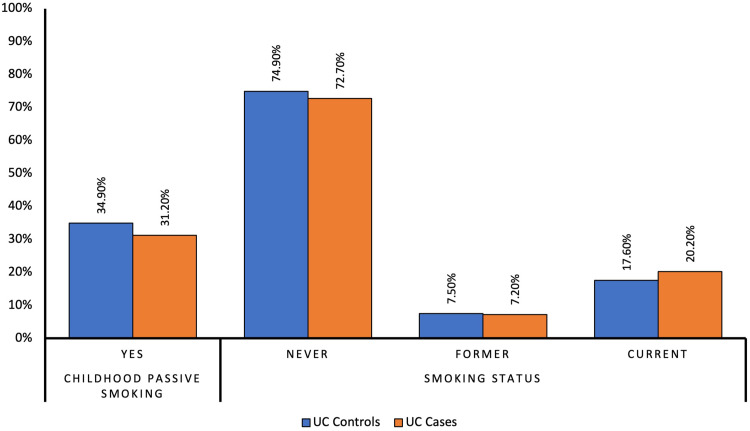
Comparison of proportions of smoking exposure between Ulcerative colitis cases and controls.

Figure 1 shows the comparison of smoking exposure between UC cases and controls. There were slightly higher proportions of controls in comparison to cases of UC who were exposed to childhood passive smoking (34.9% vs 31.2%, respectively, p=0.12), with little evidence against the null hypothesis at the study’s sample size. Similarly, higher proportions of cases with UC compared to controls were current smokers (20.2% vs 17.6%, respectively, p=0.68) and ever smokers (27.3% vs 25.1%, respectively, p=0.50) with little evidence against the null hypothesis at this sample size (Table 3). There were similar proportions of controls compared to UC cases who were former smokers (7.5% vs 7.2%, respectively, p=0.68), with little evidence against the null hypothesis at this sample size.

**Table 3:** Association Between Smoking Exposure and Ulcerative Colitis

Factor	Level	UC Controls, n (%)	UC Cases, n (%)	Univariable Analysis OR (95% CI)	*p*-value	Multivariable Analysis OR (95% CI)^a^	*p*-value
Childhood Passive Smoking	No	198 (54.8%)	194 (53.6%)	Base	Base	Base	Base
	Yes	126 (34.9%)	113 (31.2%)	0.91 (0.65–1.27)	0.578	0.86 (0.61–1.20)	0.371
Adulthood Smoking	Never	268 (74.9%)	263 (72.7%)	Base	Base	Base	Base
	Former	27 (7.5%)	26 (7.2%)	1.00 (0.57–1.78)	0.987	0.98 (0.55–1.74)	0.932
	Current	63 (17.6%)	73 (20.2%)	1.19 (0.80–1.76)	0.387	1.13 (0.75–1.69)	0.557
Ever Smoked	No	268 (74.9%)	263 (72.7%)	Base	Base	Base	Base
	Yes	90 (25.1%)	99 (27.3%)	1.13 (0.80–1.60)	0.480	1.10 (0.78–1.56)	0.590

**Note**: ^a^Adjusted for nationality and income.

**Abbreviation**: UC, Ulcerative colitis.

On multivariable analysis, compared to controls, UC cases had a 14% decrease in the odds of being exposed to passive smoking during childhood, with little evidence against the null hypothesis at this sample size (OR 0.86, 95%Cl 0.61–1.20, p=0.371). Further, compared to controls, UC cases had slightly higher odds of being a current smoker (OR 1.13, 95% CI, 0.75–1.69, p=0.557) with little evidence against the null hypothesis at the current sample size, slightly higher odds of being ever smokers, again with little evidence against the null hypothesis at the study’s sample size (OR 1.10, 95% CI, 0.78–1.56, p=0.590), and almost similar odds of being former smokers (Table 3).

Subgroup analysis by gender showed that compared to controls, male UC cases had slightly higher odds of being current smokers and of being ever smokers, although the p-value suggested a lack of statistical significance (Supplementary Table S1). However, in females, there was no association between all the smoking variables and UC (Supplementary Table S1).

### The Association Between Smoking Exposure and Crohn’s Disease

**Figure 2:**
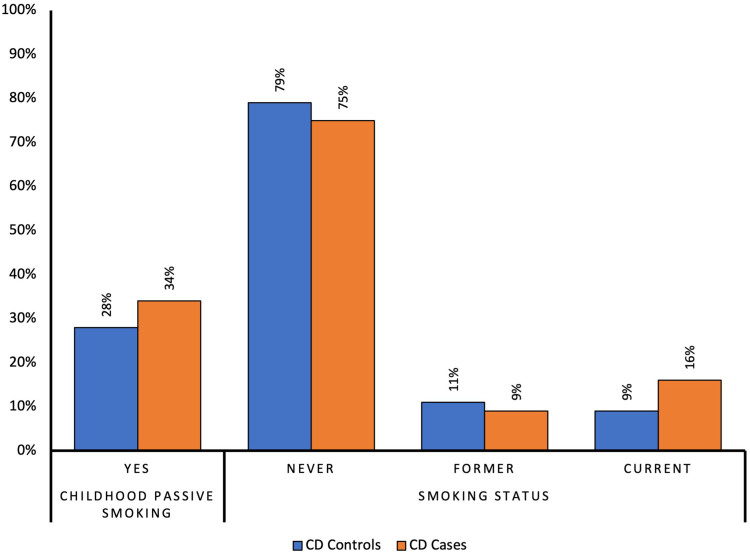
Comparison of proportions of smoking status between Crohn’s disease cases and controls.

There were slightly higher proportions of CD cases in comparison to controls who were exposed to childhood passive smoking (34% vs 28%, respectively, p=0.71) with little evidence against the null hypothesis at this sample size, and higher proportions of CD cases were current smokers compared to controls (16% vs 9%, respectively, p=0.39), again with little evidence against the null hypothesis at the study’s sample size (Figure 2 and Table 4).

**Table 4:** Association Between Smoking Exposure and Crohn’s Disease

Factor	Level	CD Cases, n (%)	CD Controls, n (%)	Univariable Analysis OR (95% CI)	*p*-value	Multivariable Analysis OR (95% CI)^a^	*p*-value
Childhood Passive Smoking	No	49 (55%)	46 (52%)	Base	Base	Base	Base
	Yes	25 (28%)	30 (34%)	1.23 (0.66–2.30)	0.517	1.18 (0.59–2.36)	0.641
Adulthood Smoking	Never	69 (79%)	67 (75%)	Base	Base	Base	Base
	Former	10 (11%)	8 (9%)	0.78 (0.27–2.25)	0.642	0.99 (0.30–3.24)	0.981
	Current	8 (9%)	14 (16%)	1.69 (0.67–4.31)	0.269	2.51 (0.85–7. 37)	0.095
Ever Smoked	No	69 (79%)	67 (75%)	Base	Base	Base	Base
	Yes	18 (21%)	22 (25%)	1.21 (0.60–2.46)	0.591	1.33 (0.63–2.83)	0.453

**Note**: ^a^Adjusted for nationality, income, and education.

**Abbreviation**: CD, Crohn’s disease.

After multivariable logistic regression, compared to controls, CD cases had 1.18 greater odds of being exposed to passive smoking during their childhood (OR 1.18, 95% CI 0.59–2.36, p=0.641) with little evidence against the null hypothesis at this sample size. Adults with CD had 2.51 times greater odds of being a current smoker compared to controls, (OR 2.51, 95% CI 0.85–7.37, p=0.095), with weak evidence against the null hypothesis at the study’s sample size. Further, CD cases had a 33% increase in the odds of being ever-smokers compared to controls, (OR 1.33, 95% CI, 0.63–2.83, p=0.453) (Table 4), also with little evidence against the null hypothesis at this sample size.

In subgroup analyses, in females, CD was significantly associated with childhood passive smoking exposure (OR 8.23, 95% CI, 1.36–49.66, p=0.021), significantly associated with adulthood current smoking (OR 42.71, 95% CI, 1.17–1559.57, p=0.041), and showed a possible association with ever smoking (OR 2.23, 95% CI 0.54–9.22, p= 0.268), although the later association showed little evidence against the null hypothesis, suggesting a lack of statistical significance. Notably, the estimates of the OR were imprecise, shown by the wide 95% CIs, because of the small sample size of women (n=80), and the low proportion of smokers in women (6% current smokers, 29% exposed to childhood smoking). In males, childhood smoking exposure showed a weak inverse association with CD, with little evidence against the null hypothesis, while weak non-significant associations were also observed between CD and both current and ever smoking (Supplementary Table S2).

## Discussion

In this matched case-control study of participants in the QBB, we found that adulthood tobacco smoking was associated with higher odds of CD, although with weak evidence against the null hypothesis at the study’s sample size, indicating weak statistical significance. Further, we found no associations between childhood passive tobacco smoking exposure and CD, and no associations between both childhood and adulthood smoking exposure and UC. In subgroup analyses, we found that both childhood smoking exposure and adulthood current smoking were strongly associated with higher odds of CD in women but not in men.

We found that UC cases had a small, 14%, decrease in the odds of being exposed to childhood passive tobacco smoking and a very small increase in the odds of being adult smokers. However, these associations were neither clinically nor statistically significant, and therefore suggest no real association between either childhood passive or adult active tobacco smoking exposure and UC development in this population. The findings of this study on adult active smoking are unlike most findings from observational studies, where adult smoking appears to confer protection against UC development.^9^ Notably, although most literature points towards a strong protective role for smoking in UC, similar to our findings, there are a few studies which have also reported no associations between adult smoking and UC.^19^,^20^ This suggests there is still a need to explore this association further, perhaps using objective smoking exposure measures. On the other hand, our findings that childhood passive tobacco smoking is not associated with UC is consistent with existing research, where childhood passive smoking exposure has not been found to affect the risk of UC in 13 different studies.^8^ It is still not clear why, in many studies, smokers and especially former smokers, have protection against UC. Further, attempts to isolate the components of tobacco smoke that are beneficial against UC have been unsuccessful. Apart from possible differences in the effect of tobacco smoke across populations on IBD, another possible explanation for the dissimilarity between our study and the other published studies could be because of the retrospective self-reported measurements of smoking in the parent QBB study. Prospective studies where objective assessments of smoking such as serum cotinine and the use of validated questionnaires are needed in the MENA setting. However, the deleterious effects of tobacco smoking on general health and its association with increased risk of many diseases is well documented, and is supported by these findings which suggest that tobacco smoking plays no protective role in UC risk in this population.

In the current study, CD was associated with a clinically significant two-and-a-half-fold increase in the odds of being a current smoker although with weak evidence against the null hypothesis at the study’s sample size, suggesting a lack of statistical significance. The higher odds ratios in current smokers that were observed in the study are consistent with other studies,^11^,^21–24^ including a meta-analysis,^9^ which indicate current smokers have an elevated risk of CD, except for the previously mentioned studies in Spain and amongst Ashkenazi Jews.^11^,^12^ The weak evidence against the null hypothesis in the study is likely due to a lack of power, as there were only 89 cases identified in the QBB database and included in this study. Further, our finding of neither a clinically nor statistically significant association between childhood passive tobacco smoking exposure and CD is consistent with existing research, where childhood passive smoking exposure has been consistently found to have no association with CD.^8^,^25^,^26^ Overall, this study suggests a detrimental effect of adult smoking on the risk of CD in a previously understudied population in the Middle East. These findings, together with the existing literature, suggest a need for smoking cessation initiatives in populations at high risk of CD in the region, where smoking is prevalent, particularly amongst men, for whom it is culturally acceptable.

Our subgroup analysis suggested that both childhood smoke and adulthood tobacco smoke exosure significantly increased the odds of CD in female smokers, but no effect was observed in males. These findings support previous research from France, where smoking was shown to increase the risk of earlier CD and worse clinical outcomes in women but not in men.^7^ A limitation of our subgroup analysis was the small sample size of women with CD and the low prevalence of female smokers in our sample, and therefore these findings require cautious interpretation. Although we observed no association between UC and smoking variables in women, similar to the French study,^7^ our findings differ in that, in men we did not observe the protective effect that was reported in the study from France.^7^ More research is needed to understand why gender appears to modify the association between tobacco smoking and IBD.

The pathways by which cigarette smoke has divergent effects in UC and CD are not yet well understood. This may be due to the large number of components of cigarette smoke and the many effects they have on the body. The contents of cigarettes, namely nicotine and carbon monoxide, are known to cause alterations to different cellular and tissue functions, for example the immune system, mucosal barriers, intestinal microbiome, and vasculature.^27^ Nicotine may provide anti-inflammatory effects by increasing mucin synthesis, reducing the expression of IL-8 by acting on nicotinic acetylcholine receptors, and reducing the production of TNF-alpha.^27^ In CD, an increase in carbon monoxide concentration has been shown to exacerbate the capacity of vasodilation in chronically inflamed micro vessels, which leads to ischemia and perpetuates ulceration and fibrosis.^27^

The present study has some limitations. First, data on duration of smoking, smoking quantities and IBD disease duration were not available. Second, the data pertaining to childhood and adulthood exposures may be subject to recall imprecision due to the long time between exposure and its measurement. This could apply equally to cases and controls because the participants were not aware of the current study hypothesis when the data were collected, and therefore may bias the odds ratios towards the null. Objective measurements such as serum cotinine in longitudinal cohorts or more robust smoking assessment tools may provide better quality data in future research and may yield important insights regarding the development and prognosis of IBD in this region.

## Conclusion

In this Middle Eastern population, adulthood smoking appears to be associated with increased odds of CD while there seems to be no association between either childhood or adulthood tobacco smoking exposure and the odds of UC. Further, our findings suggest that both childhood and adulthood cigarette smoke exposure may possibly have a detrimental effect on the odds of CD in females but not in males, although further research is needed.
